# Exploring the Needs of Spousal, Adult Child, and Adult Sibling Informal Caregivers: A Mixed-Method Systematic Review

**DOI:** 10.3389/fpsyg.2022.832974

**Published:** 2022-03-25

**Authors:** Srishti Dang, Anne Looijmans, Giulia Ferraris, Giovanni Lamura, Mariët Hagedoorn

**Affiliations:** ^1^Department of Health Psychology, University Medical Center Groningen, University of Groningen, Groningen, Netherlands; ^2^Centre for Socio-Economic Research on Ageing, INRCA IRCCS, National Institute of Health and Science on Ageing, Ancona, Italy

**Keywords:** informal caregiver, care recipient, needs, relationships, systematic review

## Abstract

Informal caregivers (ICGs) provide care to their family or friends in case of an illness, disability, or frailty. The caregiving situation of informal caregivers may vary based on the relationship they have with the care recipient (CR), e.g., being a spouse or being an adult child. It might be that these different ICGs also have different needs. This study aims to explore and compare the needs of different groups of ICGs based on the relationship they have with their CR. We conducted a systematic review, performing a search in the databases PubMed, CINAHL, and PsycINFO. We included studies with qualitative, quantitative, or mixed-method study designs. We analyzed the data using the thematic analysis method. We included 22 articles (18 qualitative; 4 quantitative). The included articles reported the needs of ICGs taking care of a spouse (spousal ICGs), parent (adult child ICG), or sibling aged 18 years or above (adult sibling ICGs). We did not include other relationships due to the limited number of articles on these relationships. The most prominent needs reported by the spousal, adult child, and adult sibling ICGs were the need for information and need for support. The three groups differed in their needs as well. Adult child and adult sibling ICGs indicated a need to be acknowledged by the people around them for their role of carer, while they also needed to be seen as an individual having their own personal needs. Moreover, spousal ICGs indicated a unique need of redefining their role and relationship with their CR. Overall, the findings indicate that along with experiencing common needs, the investigated groups have unique needs as well. Knowing the needs of different groups of ICGs can help develop tailored solutions to improve the quality of life of the ICGs and their CR.

**Systematic Review Registration:** [www.crd.york.ac.uk/prospero/], identifier [CRD42020188560].

## Introduction

With the gradual increase in life expectancy and longer periods of disability and chronic illness in people’s lifetime, there is increasing demand for informal caregivers (ICGs) in the health, social, and long-term care systems (Colombo and Mercier, 2012). ICGs provide unpaid assistance or care to a person with frailty, a chronic illness, or a disability (Roth et al., 2015). They assist the care recipient (CR) with activities such as bathing, clothing, shopping, cooking, household chores, and managing finances (Roth et al., 2015; Schwartz et al., 2021). Along with professional caregivers, ICGs form a major pillar of the health, social, and long-term care systems in any country (Yghemonos, 2016).

Spousal ICGs are often co-residing with the CR and are considered in most cases the primary caregivers (Tennstedt et al., 1993). They are primarily responsible for household tasks and provide more hours of caregiving than adult child ICGs as they live with the CR (Tennstedt et al., 1993; Pinquart and Sörensen, 2011). Moreover, spousal ICGs may receive less support from family and friends as compared to adult child ICGs (Pinquart and Sörensen, 2011). In contrast, adult child ICGs most often do not live with the CR and may have a choice to decide whether they want to provide care or not (Schulz et al., 2012). They often combine caregiving with other roles in life, such as being a student or employee, and therefore find it challenging to balance caregiving with other activities (Stephens et al., 2001; Broese van Groenou et al., 2013; Bastawrous et al., 2015). ICGs providing care to, for example, their sibling or grandparent, are most often considered secondary ICGs and assist the primary ICGs (i.e., spousal or adult child ICGs). They usually perform less intense care and contribute fewer hours in caregiving (Barker, 2002; Egging et al., 2011).

Caregiving may be challenging (Buchanan et al., 2013; Tan et al., 2018). Due to the demanding care responsibilities, ICGs can experience negative physical (e.g., fatigue and pain affecting daily activities) and psychological problems (e.g., stress and anxiety) (Do et al., 2015; Goren et al., 2016; de Zwart et al., 2017; Hansen et al., 2021). Spousal ICGs have been found to experience a higher overall subjective burden, financial burden, and more physical and mental health problems than adult child ICGs (Pinquart and Sörensen, 2011; Oldenkamp et al., 2016). Various studies have suggested that poor physical and psychological outcomes may be a consequence of insufficient support and unmet needs experienced by the ICGs (Etters et al., 2008; Tatangelo et al., 2018). Therefore, one way to reduce the burden among the ICGs may be to understand the unmet needs and provide ICGs with appropriate support.

In the literature, the need for information and the need for support are expressed as the most prominent needs by ICGs (Wang et al., 2018). Studies have reported that ICGs wished to be informed about various topics ranging from knowing about the CR’s health condition to information concerning service availability (Docherty et al., 2008; Washington et al., 2011; Gillespie et al., 2021). They also expressed a need for support from family members, friends, and health care workers (Mollica et al., 2020). Furthermore, ICGs expressed needs at the personal level, where they needed time for themselves and to take care of their own health (Tatangelo et al., 2018; Akgun-Citak et al., 2020).

Since the caregiving situation of ICGs can vary based on the relationship they have with the CR, e.g., being a partner or being an adult child, it could be the case that different groups of ICGs also have different needs. As per our knowledge, there is no overview of the literature that compares the needs of several groups of ICGs based on the relationship they have with the CR. Therefore, this study aims to provide a systematic overview of the existing literature on the needs of specific ICGs groups based on their relationship with the CR. Knowledge about the common and unique needs of different groups of ICGs may help health care professionals to provide tailored support to ICGs that could help reduce ICGs’ burden. Moreover, the findings have the potential to help develop and implement solutions that are tailored to the unique needs of the ICGs based on their relationship with the CR.

## Methods

### Search Strategy

We conducted a systematic review following the Preferred Reporting Items for Systematic Reviews and Meta-Analysis (PRISMA) guidelines (Page et al., 2021). The protocol of this systematic review has been registered in the International Prospective Register of Systematic Reviews (PROSPERO ID: CRD42020188560). The University Medical Center Groningen (UMCG) team (TI, AL, MH, and SD) established the search strategy. A senior researcher (JW) from Uppsala University then reviewed it following the PRESS peer review guidelines (McGowan et al., 2016). SD searched the electronic databases PubMed, CINAHL, and PsycINFO) in October 2019 for publications in the period of January 2010 to October 2019. Later the search was extended till November 2021. We created a search string using the free text words and MESH terms when available. We combined the three main categories with the Boolean operator “AND” to identify the relevant articles. The categories were: the informal caregiving population (e.g., caregiving, caring), needs (e.g., needs, needs assessment), and family relationships (e.g., spouses, children). We used the following search string in the PubMed database:

((((“Caregivers”[Mesh] OR caregiv*[tiab] OR ((“Family”[Mesh] OR family[tiab] OR spous*[tiab] OR parent*[tiab] OR husband*[tiab] OR wife[tiab] OR wives[tiab] OR partner*[tiab] OR adult child*[tiab]) AND caring[tiab])))) AND ((“Health Services Needs and Demand”[Mesh] OR “Needs Assessment”[Mesh] OR needs[tiab]))) AND ((“Spouses”[Mesh] OR “Siblings”[Mesh] OR wife[tiab] OR wives[tiab] OR husband*[tiab] OR marital[tiab] OR spous*[tiab] OR sibling*[tiab] OR adult child*[tiab] OR brother*[tiab] OR sister*[tiab] OR daughter*[tiab] OR son[tiab] OR sons[tiab] OR granddaughter*[tiab] OR grandson*[tiab] OR grandchild*[tiab])).

In addition to the advanced search on the electronic databases, SD performed a reference check of the final shortlisted articles to identify the relevant studies that were not found in the database search.

### Selection of Studies

Two authors, SD and GF, independently performed the selection of studies in two phases. In the first phase, the titles and abstracts of all articles found in the electronic database search were screened. The author (GF) screened 10% of the retrieved articles in the first phase (Gough et al., 2017). To be included, studies needed to meet the following criteria: (i) they provided data on the needs or unmet needs of one or multiple groups of informal caregivers, i.e., ICGs taking care of a spouse, parent, adult child, adult sibling, grandparent or grandchild; (ii) had a qualitative, quantitative or mixed-method study design, (iii) were published in English; and (iv) included adult ICGs, i.e., aged 18 years or above. Studies were excluded if (i) they presented outcomes in which data of one group of ICGs were mixed with the data of another group of ICGs or (ii) were published as gray literature (i.e., conference abstracts, presentations, proceedings; unpublished trial data; government publications; and reports such as white papers, working papers, and internal documentation).

In the second phase, we screened the shortlisted articles for full text. The author (GF) again screened 10% of the shortlisted articles in the second phase. The definition of need was kept broad, including personal (e.g., need for leisure time) and care-related needs (e.g., need for caregiving support) of the ICGs. Articles describing an evaluation of an intervention program (i.e., describing what ICGs need in that specific program) were not considered. In this study, we define spousal ICGs as caregivers who were in an intimate relationship with their CR; they could be either married, living in a partnership, or unmarried, irrespective of whether they share the same household or not. The studies were excluded when both authors (SD and GF) were convinced that the study did not meet the inclusion criteria. Authors SD and GF experienced disagreement in 5% of the total number of articles screened by GF in the first phase and 2% in the second phase. These discrepancies were resolved with a detailed discussion between the researchers. If no consensus was reached, the articles were screened and discussed by the other authors to reach a consensus.

### Data Extraction

SD and GF independently extracted the data from the selected studies. The researcher (GF) extracted the data from one-third of the selected studies. The following data were extracted and recorded in a table: author name, year of publishing, country, study aim, study design, sample characteristics, the relationship of the ICGs with their CR, illness of the CR, and type of needs expressed by the ICGs (Supplementary Table 1). We developed the table based on the Cochrane data collection form for intervention reviews on randomized controlled trials (RCTs) and non-RCTs (Li et al., 2021).

### Quality Assessment of Studies

The quality assessment was performed by two authors, SD and GF independently for all the 22 selected studies. After completing the phase both the authors discussed the discrepancies in selecting the studies. The discrepancies were resolved with a detailed discussion between the researchers. If no consensus was reached, the articles were screened and discussed by the other authors (MH and SD) to reach a consensus. The quality of included qualitative studies was accessed using the Critical Appraisal Skills Program checklist (CASP) (CASP, 2017). The checklist consists of 10 questions, which assess qualitative studies on the following criteria: clarity of the aim, appropriateness of the study design, and the validity and implications of the findings. The first two questions were the screening questions, and if the answer to both was “yes,” the author proceeded with the remaining questions. All the questions were evaluated using the parameters “yes,” “no,” or “cannot tell.” “Yes” corresponded to strong quality, “no” to moderate, and “cannot tell” to weak quality with respect to the specific criteria (Table 1).

**TABLE 1:** Quality assessment of qualitative studies according to the Critical Appraisal Skills Program (CASP).

Authors	Clear statement of the aims of research	Appropriateness of qualitative methodology	Appropriateness of research design	Appropriateness of recruitment strategy	Appropriateness of data collection strategy	Relationship considered between research and participants	Ethical issues considered	Data analysis rigorous	Clear statement of the findings	Value of the research
Andela et al. (2019)	Yes	Yes	Yes	Yes	Yes	Can’t tell	Can’t tell	Yes	Yes	Yes
Badr et al. (2016)	Yes	Yes	Yes	Yes	Yes	Can’t tell	Yes	Yes	Yes	Yes
Evertsen and Wolkenstein (2010)	Yes	Yes	Yes	Can’t tell	Yes	Yes	Yes	Yes	Yes	Yes
Habermann and Shin (2017)	Yes	Yes	Yes	Yes	Yes	Can’t tell	Can’t tell	Yes	Yes	Yes
Hupcey et al. (2011)	Yes	Yes	Yes	Can’t tell	Yes	Can’t tell	Can’t tell	Yes	Yes	Yes
Johannessen et al. (2017)	Yes	Yes	Yes	Yes	Yes	Can’t tell	Yes	Yes	Yes	Yes
Le Dorze and Signori (2010)	Yes	Yes	Yes	Yes	Yes	Can’t tell	Can’t tell	Yes	Yes	Yes
Morrisby et al. (2019)	Yes	Yes	Yes	Yes	Yes	Can’t tell	Yes	Yes	Yes	Yes
Wawrziczny et al. (2017)	Yes	Yes	Yes	Yes	Yes	Can’t tell	Yes	Yes	Yes	Yes
Barca et al. (2014)	Yes	Yes	Yes	Yes	Yes	Can’t tell	Can’t tell	Yes	Yes	Yes
Nicholls et al. (2017)	Yes	Yes	Yes	Yes	Yes	Can’t tell	Yes	Yes	Yes	Yes
Figueiredo et al. (2016)	Yes	Yes	Yes	Yes	Yes	Can’t tell	Yes	Yes	Yes	Yes
Tatangelo et al. (2018)	Yes	Yes	Yes	Yes	Yes	Yes	Yes	Yes	Yes	Yes
Arnold et al. (2012)	Yes	Yes	Yes	Yes	Yes	Can’t tell	Can’t tell	Yes	Yes	Yes
Amaresha et al. (2015)	Yes	Yes	Yes	Yes	Yes	Can’t tell	Yes	Yes	Yes	Yes
Davys et al. (2016)	Yes	Yes	Yes	Yes	Yes	Can’t tell	Yes	Yes	Yes	Yes
Grant et al. (2021)	Yes	Yes	Yes	Yes	Yes	Yes	Yes	Yes	Yes	Yes
Yang et al. (2017)	Yes	Yes	Yes	Yes	Yes	Yes	Can’t tell	Yes	Yes	Yes

The quality of included quantitative studies was assessed using the Quality Assessment Tool for Quantitative Studies, recommended by Cochrane (Higgins et al., 2011). The quantitative studies were rated on selection bias, study design, confounders, blinding, data collection method, and withdrawals and dropouts (Table 2), using scores from one to three, where one indicated strong, and three indicated weak quality. Based on these criteria, the overall quality of the quantitative articles was rated as “1” (strong), if there were no weak ratings; as “2” (moderate), when one component had a weak rating; and as “3” (weak) when two or more components had weak ratings.

**TABLE 2:** Quality assessment of quantitative studies according to the Quality Assessment Tool for Quantitative Studies, recommended by Cochrane.

Author(s)	Selection bias	Study design	Confounders	Blinding	Data collection method	Withdrawals and dropouts	Overall quality
Kobayakawa et al. (2016)	2	1	1	2	3	4	2
Turner et al. (2013)	2	1	1	3	1	2	2
Veil et al. (2013)	3	1	3	2	3	4	3
Peeters et al. (2010)	2	1	3	2	3	4	3

*The scoring is as following: 1, strong; 2, moderate; 3, weak; 4, not applicable.*

### Data Synthesis

We used thematic analysis, a technique of narrative synthesis (Braun and Clarke, 2006), to synthesize the data for both qualitative and quantitative studies. The thematic analysis technique is commonly used for analyzing qualitative data but could also be used for quantitative data (Popay et al., 2006). Since the majority of articles provided qualitative data, thematic analysis was an appropriate method to describe and compare the main findings. We followed the convergent integrated mixed-method approach for synthesis and integration of qualitative and quantitative data (Stern et al., 2020). We carried out three steps for the thematic analysis. In the first step we performed data transformation for quantitative studies where we extracted data from quantitative studies and converted it into textual descriptions to allow integration with qualitative data. We then extracted line by line textual data for each included qualitative study article as well. The textual descriptions (qualitized data) from quantitative studies were assembled and pooled with the qualitative data extracted directly from qualitative studies. In the second step, the authors (SD, AL, GF, and MH) familiarized themselves with the extracted data on the needs of ICGs. They generated an initial list of codes for different types of needs from the data. In the third step, when all the data was coded, initial codes were combined into themes. We used a mind map to group the codes with different types of needs into main themes of needs. The themes were compared to identify overlapping and unique needs of spousal, adult child, and adult sibling ICGs.

## Results

### Selection of Studies

**FIGURE 1:**
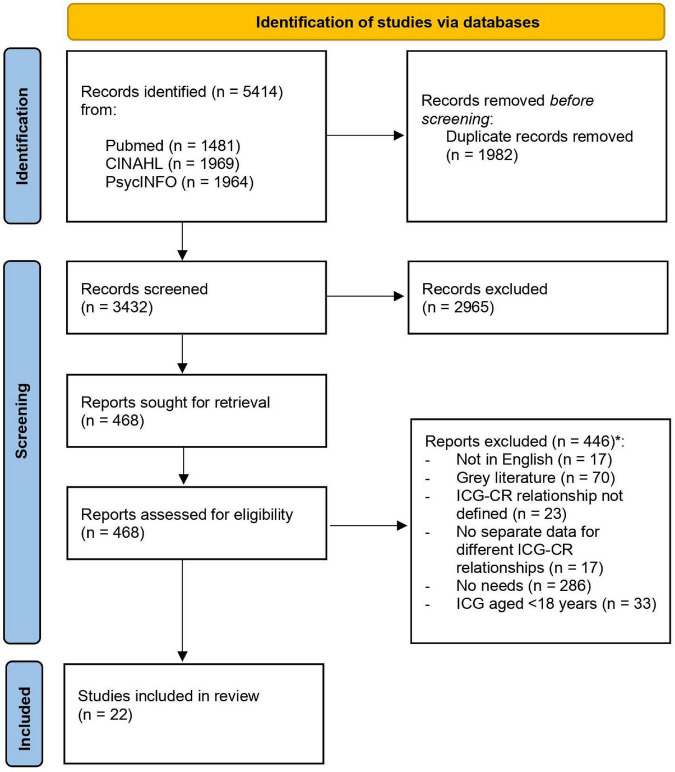
Preferred Reporting Items for Systematic Reviews and Meta-analyses (PRISMA) flow diagram of study selection process. *The reports were matched with the inclusion and exclusion criteria in the order presented here. In case a report did not meet a specific exclusion or inclusion criteria it was immediately excluded and not matched for the other criteria. For example, if the article was not published in the English language, it was excluded without checking for other criteria.

Figure 1 presents the PRISMA flowchart, reflecting the number of included studies at each step. In total, 5,414 articles were retrieved from the electronic databases, of which 468 articles were reviewed in full-text. After reviewing the full-text articles, 22 studies were included for the analysis. No new articles were included based on the reference check of the included articles.

### Study Characteristics

Supplementary Table 1 presents the characteristics and main findings of the included qualitative (*n* = 18) and quantitative (*n* = 4) studies. The included articles reported the needs of spousal ICGs, adult child and adult sibling ICGs aged 18 years or above. We found a limited number of articles that represented the relationships of parents, grandparents, and grandchildren; therefore, we did not include other relationships. Out of these 22 studies, (i) 11 articles reported the needs of spousal ICGs, (ii) five articles reported the needs of adult sibling ICGs (iii) three articles reported the needs of adult child ICGs, and (iv) three articles discussed the needs of both spousal and adult child ICGs with data analyzed separately. The sample sizes ranged from 6 to 139 ICGs for the qualitative studies and 68 to 862 ICGs for the quantitative studies. The studies were conducted in Australia, Canada, China, Denmark, France, India, Japan, Norway, Portugal, the Netherlands, United Kingdom, and United States.

### Quality Assessment

The results of the quality assessment of qualitative studies are presented in Table 1. The assessment indicates that all the studies included clear aims, qualitative methods, recruitment strategies, data collection methods, and statements of findings (18/18). According to the information reported in the articles, most of the studies had considered ethical issues (11/18) and conducted rigorous data analysis (16/18). Concerns were noted in the potential for bias in the relationship between the researcher and participants as these articles did not report on the researcher’s role and it’s influence during data collection, with only 4/18 studies meeting the CASP criteria. The results of the quality assessment of quantitative studies are presented in Table 2. The assessment indicates that only the study design component was reported strong for all the articles, whereas other components were weak for one or more studies. Based on the assessment of all the components, two of the four quantitative studies were assessed as having moderate quality (Turner et al., 2013; Kobayakawa et al., 2016), and two have a weak quality (Peeters et al., 2010; Veil et al., 2013).

The assessment was conducted to improve transparency in the systematic review process. No study was excluded based on the evaluation.

### Overall Synthesis of the Included Studies

The synthesis of the 22 included qualitative and quantitative articles resulted in seven themes of needs (Table 3). Out of the seven themes of needs, (i) four themes were reflected by all the three groups, i.e., a need for information, for support, for personal time, and to manage personal concerns, (ii) two themes were expressed only by the adult child and adult sibling ICGs, i.e., a need to be acknowledged and to be more than a carer, and (iii) one theme was expressed only by spousal and adult sibling ICGs, i.e., a need to maintain the relationship with the CR. The seven themes included certain sub-themes, which differed further for the spousal, adult child, and adult sibling ICGs. We will discuss the common and unique themes and sub-themes in detail below for the qualitative studies and quantitative studies.

**TABLE 3:** Overview of common and unique needs experienced by spousal, adult child and adult sibling ICGs.

Themes	Sub-themes	Spousal	Adult child	Adult sibling
Need for information	Information about the illness and treatment	✓	✓	✓
	Information about the CR’s health condition	✓	✓	✓
	Information about the service availability	✓	✓	✓
	Information about the role of caregiver	✓		
Need for support	Social support from family and friends	✓	✓	✓
	Supportive care from professionals	✓	✓	✓
	Financial support	✓		✓
Need for personal time	Relax and pursue leisure activities	✓	✓	✓
	Time for socializing	✓	✓	
Need to manage personal concerns	Manage physical and mental health	✓	✓	✓
	Addressing the fear of heredity of CR’s illness	✓	✓	✓
Need to maintain their relationship with the CR	Need to maintain healthy communication with the CR	✓		✓
	Redefine their role and relationship with the CR	✓		
Need to be more than a carer	Detach themselves from unexpected responsibilities of caregiving		✓	
	Nurture their personal needs		✓	✓
Need to be acknowledged	Appreciated and empathized by the family members and peer group		✓	
	Included by family and health care workers in making decisions for the CR		✓	✓

### Synthesis of Qualitative and Quantitative Studies

#### Need for Information

The most prominent need expressed by the spousal, adult child, and adult sibling ICGs was the need for more information in both qualitative (Evertsen and Wolkenstein, 2010; Le Dorze and Signori, 2010; Hupcey et al., 2011; Arnold et al., 2012; Barca et al., 2014; Amaresha et al., 2015; Badr et al., 2016; Davys et al., 2016; Figueiredo et al., 2016; Habermann and Shin, 2017; Johannessen et al., 2017; Nicholls et al., 2017; Wawrziczny et al., 2017; Andela et al., 2019) and quantitative studies (Peeters et al., 2010; Turner et al., 2013; Veil et al., 2013). Four specific domains of information were identified, which are described below.

##### Information About the Illness and Treatment

Spousal, adult child, and adult sibling ICGs indicated a need for information about the disease or illness in general (Barca et al., 2014; Amaresha et al., 2015; Figueiredo et al., 2016; Johannessen et al., 2017; Wawrziczny et al., 2017; Andela et al., 2019), its treatment (Hupcey et al., 2011; Wawrziczny et al., 2017), prognosis (Peeters et al., 2010; Amaresha et al., 2015) and how they can cope with the CR’s symptoms (Barca et al., 2014; Figueiredo et al., 2016; Wawrziczny et al., 2017; Andela et al., 2019). The articles represented a mix of CRs’ illnesses, including both physical illnesses, such as advanced heart failure (Hupcey et al., 2011) and mental illnesses, such as schizophrenia (Amaresha et al., 2015).

Adult child ICGs taking care of patients with a chronic illness indicated a need for information about the long-term implications of the illness so that they can gain control over the unpredictable nature of the illness (Nicholls et al., 2017). Moreover, spousal ICGs taking care of advanced heart failure patients needed information that can help them in making decisions both in case of an emergency or when the CR is stable (Hupcey et al., 2011).

##### Information About Care Recipient’s Health Condition

All three groups of ICGs expressed a need to be informed about the health condition of the CR (Le Dorze and Signori, 2010; Nicholls et al., 2017; Grant et al., 2021). They wanted to be involved in the information process concerning the treatment of their CR (Nicholls et al., 2017; Andela et al., 2019; Grant et al., 2021). In addition, spousal ICGs needed information about the severity of the physical symptoms of the CR (Badr et al., 2016). They expected the health care workers to use less jargon and provide easily understandable information about their CR’s condition (Le Dorze and Signori, 2010; Andela et al., 2019), keep transparency in providing a clear timeline for the recovery (Badr et al., 2016), and inform them about how long the CR’s recovery is going to be (Evertsen and Wolkenstein, 2010).

##### Information About Service Availability

Spousal (Johannessen et al., 2017; Wawrziczny et al., 2017), adult child (Veil et al., 2013), and adult sibling ICGs (Amaresha et al., 2015) indicated a need to know about the services available for them and their CRs, such as home assistance and public resources. Adult sibling ICGs needed training and programs explaining what services are available for them as an ICG and how they can avail it as most of the time, they have to seek information themselves (Arnold et al., 2012; Grant et al., 2021). Moreover, adult child ICGs taking care of CR with dementia needed information about legal amends, and help with administrative work when their CR gets admitted to the hospital (Peeters et al., 2010).

##### Information About the Role of Caregiver

Spousal ICGs expressed a need to know about their roles and responsibilities as a caregiver (Evertsen and Wolkenstein, 2010). This need was expressed only in one study focusing on female spousal ICGs taking care of a partner with prostate cancer. They expressed that they did not receive enough information before undertaking a new role of being a caregiver to their partner. They did not know what questions to ask the health care workers and had to rely on the information provided to them by the health care workers.

#### Need for Support

##### Social Support From Family and Friends

All the three groups reported a need for support from family and friends (Evertsen and Wolkenstein, 2010; Arnold et al., 2012; Amaresha et al., 2015; Nicholls et al., 2017; Wawrziczny et al., 2017; Yang et al., 2017; Andela et al., 2019; Morrisby et al., 2019). Spousal ICGs seek support from their family and friends, especially from their children, but did not want to disturb their children, as they were busy in their lives (Wawrziczny et al., 2017). Moreover, spousal ICGs experienced diminishing social relationships due to (i) practical difficulties of managing the behavior and psychological symptoms of their CR in public and (ii) a lack of understanding of other people (Tatangelo et al., 2018).

Adult child and adult sibling ICGs also experienced a lack of support from family and friends. They needed their social network to understand their efforts and struggles as caregivers (Arnold et al., 2012; Tatangelo et al., 2018). To gain support from a wider social network, they needed people, in general, to be educated about the illness of their CR, so that it can become an accepted subject to talk about (Arnold et al., 2012; Barca et al., 2014).

##### Supportive Care From Professionals

All three groups expressed a need for professional support, but the type of professional support needed varied for the three groups. Spousal ICGs mainly required respite care services such as day-care centers or assistance at home (Johannessen et al., 2017; Andela et al., 2019) or institutional support that is timely, effective, and affordable to help them in caregiving (Morrisby et al., 2019). They also indicated a need for emotional support from a mental health professional (Kobayakawa et al., 2016). Although spousal ICGs sometimes were aware of the existence of the services, they were unable to procure due to lack of quality and flexibility offered by the services or lack of support from the CR (Tatangelo et al., 2018).

Adult child and adult sibling ICGs needed a professional counselor or health care worker whom they could trust to talk to Nicholls et al. (2017). They needed more attention from the professionals, follow-up services for themselves, and a stable contact with whom they could talk in case of emergency (Peeters et al., 2010; Amaresha et al., 2015; Nicholls et al., 2017). Moreover, adult child ICGs also expected better coordination with the professionals that would help them in caregiving (Peeters et al., 2010). All three groups indicated a need for a support group of persons who are going through the same struggles and with whom they can share their experiences openly (Evertsen and Wolkenstein, 2010; Arnold et al., 2012; Barca et al., 2014; Badr et al., 2016).

##### Financial Support

Both spousal and adult sibling ICGs needed financial help and government benefits to support them in caregiving (Arnold et al., 2012; Turner et al., 2013; Habermann and Shin, 2017). They expressed a need to be educated about the financial resources available for them and how they can avail those resources (Le Dorze and Signori, 2010; Arnold et al., 2012). Spousal ICGs indicated financial hardships due to the caregiving where they experienced unmet needs regarding inadequate financial resources and the resultant financial strain (Habermann and Shin, 2017). In addition, they needed financial aid in paying for expensive treatments (Hupcey et al., 2011).

#### Need for Personal Time

All three groups indicated a need to take a break from their caregiving role. They wanted time for socializing, pursuing their leisure activities and holidays (Amaresha et al., 2015; Wawrziczny et al., 2017; Tatangelo et al., 2018). Spousal ICGs wished for time away from caregiving and wanted someone to take away the feeling that they have to be present for their CR all the time (Le Dorze and Signori, 2010; Habermann and Shin, 2017), whereas adult child ICGs perceived a conflict of time between caregiving and other activities (Tatangelo et al., 2018).

#### Need to Manage Personal Concerns

##### Manage Physical and Mental Health

All three groups expressed a need to take care of their physical and mental health. They all needed professional support to manage their mental health. A comparative study (Tatangelo et al., 2018) on sibling and adult child ICGs taking care of a CR having dementia indicated similar health needs for both groups. Although the spousal ICGs were hesitant in acknowledging their unmet health needs, adult child ICGs could easily identify their health needs. Spousal ICGs felt that their needs were secondary to the needs of the person they cared for. Both groups felt they needed more time to exercise and pursue healthy eating habits. Whereas a study on adult sibling ICGs (Amaresha et al., 2015) taking care of CR having schizophrenia indicated that they needed help managing their day-to-day stressors arising from caregiving.

##### Addressing the Fear of Heredity

Spousal ICGs taking care of CR with cancer (Turner et al., 2013), adult child ICGs taking care of CR with chronic illness (Nicholls et al., 2017), and adult sibling ICGs taking care of CR with schizophrenia (Amaresha et al., 2015) needed help in addressing the fear of the hereditary or familial risk of CR’s illness. They wanted to know about the unpredictable nature of the CR’s illness, especially where there was a chance of hereditability.

#### Need to Maintain the Relationship With the Care Recipient

##### Need to Maintain a Healthy Communication With Their Care Recipient

This need was indicated by the spousal (Badr et al., 2016) and adult sibling ICGs (Amaresha et al., 2015) taking care of a CR with head and neck cancer and schizophrenia, respectively. The participants of the two studies were either all (Badr et al., 2016) or mostly women (Amaresha et al., 2015). The sibling ICGs felt irritated with the behavior of their CR, therefore, needed tips from health care professionals to manage their communication with the CR (Amaresha et al., 2015). The spousal ICGs felt that their relationship with the CR was highly compromised during the cancer treatment due to the poor health condition of the CRs. Moreover, they struggled to get a response from the CR and had to hold back their emotions from their CR (Badr et al., 2016).

##### Redefine Their Role and Relationship With the Care Recipient

This sub-theme was indicated only by the spousal ICGs. They experienced a decrease in physical intimacy, such as kissing, hugging, touching since the treatment. Moreover, spousal ICGs did not want to disrupt the balance in their relationship by being just a ‘life coach’ for their CR (Andela et al., 2019). In one article, a spousal ICG expressed, “I wish I could do more, but we cannot. For example, I would love to go to the restaurant as we used to do before” (Wawrziczny et al., 2017). They wished to maintain (Wawrziczny et al., 2017) and redefine (Le Dorze and Signori, 2010) their relationship with their CR by pursuing activities together and creating shared moments.

#### Need to Be Acknowledged

Studies reported that both adult child and adult sibling ICGs expressed a need to be acknowledged as a caregiver by the people around them, including friends, family, and health care workers (Arnold et al., 2012; Barca et al., 2014; Nicholls et al., 2017; Grant et al., 2021). The family members and health care workers did not see them as a caregiver, therefore did not involve them in making important decisions for the CR (Nicholls et al., 2017). Moreover, adult child ICGs expressed another need, namely the need to be appreciated by the family members and peer groups for their caregiving role. They did not receive the desired appreciation and empathy from their family members (Nicholls et al., 2017).

#### Need to Be More Than a Carer

Both adult child and adult sibling ICGs struggled to take time away from caregiving for personal activities. A common need for both adult child ICGs and adult sibling ICGs was a need to be more than just a carer and to be individuals who have their own feelings and personal needs (Barca et al., 2014; Davys et al., 2016; Nicholls et al., 2017). A participant in one study (Nicholls et al., 2017) expressed it in these terms: I just wanted to escape and be by myself, thus highlighting ICGs’ need to detach themselves from unexpected caregiving responsibilities and make time to nurture their personal needs.

## Discussion

The goal of this systematic review was to explore the needs of different groups of ICGs based on the ICG-CR relationship. We compared spousal, adult child, and adult sibling ICGs to identify their common and unique needs. After performing a comprehensive search of the literature, we included 22 articles in our systematic review. The analysis showed that multiple articles from all three groups reported the need for information and need for support. All three groups needed information about the CR’s illness, health condition of their CR, and service availability such as home assistance and respite care services. All the groups also expressed a need for social support from family and friends and supportive care from professionals. Spousal and adult sibling ICGs also needed financial help to support them in caregiving. The three groups differed in their needs as well. It is noteworthy that all the qualitative articles targeting adult child ICGs and multiple articles targeting adult sibling ICGs indicated a need to be acknowledged by the people around them for their role of carer, while they also needed to be seen as an individual having their own personal needs. Moreover, multiple articles from spousal ICGs indicated a unique need of redefining their role and relationship with their CR. Before we discuss and interpret our findings, it is important to highlight possible biases that could have influenced our findings.

Out of the 22 included articles, only three articles directly compared ICG groups (i.e., spousal and adult child ICGs, but not adult sibling ICGs) (Peeters et al., 2010; Figueiredo et al., 2016; Tatangelo et al., 2018). Self-evidently, a direct comparison within the same study (i.e., same aim, study design, and procedure) would be the strongest design for determining whether different types of ICGs have common or unique needs. The lack of comparative articles means that in our analysis, we compared the needs of spousal, adult child, and adult sibling ICGs based on articles that showed heterogeneity in their study aim and study design. In some articles, reporting the needs was not the main aim of the study, and therefore, the needs were not explained in detail, or the definition of certain needs remained unclear (e.g., Wawrziczny et al., 2017; Andela et al., 2019). Moreover, the aim of the articles differed which resulted in differences in the type of needs explored or reported in the study. For example, in some articles, the aim of certain studies was directed to a specific need such as the need for support (e.g., Morrisby et al., 2019), whereas in others, all types of needs were explored (e.g., Le Dorze and Signori, 2010). The articles also differed in their study design, which influenced the type of data collected. For example, the data reported by articles with a qualitative design (e.g., focus groups) was more rich and descriptive in nature than findings of studies with a quantitative design using closed-ended questionnaires, limiting the scope to specific needs based on the aim of the study. The articles also showed heterogeneity in the study aim with respect to the illness of CR. Different illnesses (e.g., cognitive versus physical illness) may result in different needs in ICGs. However, in our outcomes, we did not find any reason to assume an association between different types of illnesses of the CR and the needs experienced by ICGs.

In addition, articles differed in the demographic characteristics of the participating ICGs, which could again influence the needs. For example, the needs of younger ICGs may differ from those of older ICGs, or the needs of ICGs residing with the CR may differ from those living far away. Thus, considering the scarcity of studies that directly compare different types of ICGs and the heterogeneity in the articles included, cautiousness in drawing conclusions is needed. Nevertheless, when we looked at the articles directly comparing the spousal and adult child ICGs, their findings seem to be in line with those of articles studying individual groups of ICGs. For example, the comparative study by Tatangelo et al. (2018) reported that spousal ICGs needed personal time away from the caregiving role, which is similar to the findings by Wawrziczny et al. (2017), where spousal ICGs needed time for socializing and pursue their leisure activities.

### Need for Information

All three groups indicated the need to know about the availability of services. This need was expressed by spousal and adult sibling ICGs in a qualitative study, and by adult child ICGs in a quantitative study, therefore the service needs for spousal and adult sibling ICGs were more descriptive in nature. They indicated the type of services they needed, such as home assistance and respite care, whereas the adult child ICGs only indicated a need to know about the services available and where to find them. The literature indeed suggests that ICGs lack awareness about the availability of services (Wiles, 2003; Bieber et al., 2019), and this lack of awareness acts as an important barrier that withholds ICGs to access and use these formal services and support (Innes et al., 2011; Bieber et al., 2019). Other barriers that withhold ICGs from accessing the services are lack of flexibility of services, lack of availability, or lack of support from the CR especially for respite care services (Fine and Thomson, 1997). It is important for ICGs to know about the availability of services as the literature suggests that ICGs who are unable to use the formal services and support often experience high levels of caregiver burden and poor health outcomes (Fine and Thomson, 1997).

### Need for Support

All three groups expressed a need for social support from people around them and supportive care from the professionals. Interestingly, spousal and adult sibling ICGs expressed an additional need for financial support, which was not indicated by adult child ICGs. This need is in line with the literature that spousal ICGs experience higher financial strains than adult child ICGs and other relationships (Van Houtven et al., 2010; Pinquart and Sörensen, 2011; Lee and Zurlo, 2014). This could be because of the financial interdependence in a marital relationship, where the financial responsibility has to be taken care of by ICGs due to the illness of their spouse. Interestingly, adult sibling ICGs also expressed a need for financial support. There is limited literature to support the findings, but it could be linked to their transition from secondary to the primary caregiver in the later stage of life, where they express a need to be included in future planning for financial matters (Taggart et al., 2012; Davys et al., 2016).

### Need to Be Acknowledged

The need to be acknowledged was expressed only by the adult child and adult sibling ICGs. Our findings are in line with the literature that suggests that spousal ICGs by default are acknowledged as caregivers by society because of the nature of their relationship with the CR (Lee and Smith, 2012). Adult child ICGs have the experience of caregiving, which is not recognizable by their peer group or family members, making it difficult for them to be understood or appreciated by the people around them (Nicholls et al., 2017). Adult siblings are not considered traditional caregivers, and their struggle of not being involved in caregiving by family members and health care workers is well discussed in the literature (Burke et al., 2015). They want to be involved by family members in future planning, especially in plans related to finance and legal matters (Heller and Arnold, 2010).

Adult child ICGs expressed an additional need to detach themselves from the responsibilities of caregiving. The two articles that indicated this need included a population of mostly young adult caregivers (YACs), the age of these ICGs ranged from 18 to 30 years. As we know from previous literature, young adults in this age group are in their transition period between being young and at the same time entering adulthood, establishing themselves with respect to certain aspects of life such as education, career, relationships, and social life. This period may be more challenging for YACs who have to integrate caregiving with other aspects of life (Haugland et al., 2020). We know from literature that caregiving has an impact on YAC’s academic performance, they get fewer opportunities to connect with their peers in college, maintain their relationships with friends and close ones, or start new relationships (Mickens et al., 2017). Therefore, it is imaginable that YACs experience a need to allow themselves to be young adults without thinking about caregiving responsibilities (Stephens et al., 2001; Broese van Groenou et al., 2013; Bastawrous et al., 2015).

## Limitations, Strengths, and Future Recommendations

We noticed several strengths and limitations of our review. Our review explored the needs of all the ICG-CR relationships in the literature, such as grandparental ICGs or extended family ICGs. After an extensive search of the literature, we ended up with multiple articles for spousal, adult child and adult sibling ICGs. Another strength is that all the included articles were checked for their quality using validated and standardized measures. Lastly, we pre-registered our review in PROSPERO before conducting it, thus, fostering the transparency of the systematic review process (Schiavo, 2019). A limitation of this review is that articles in languages other than English were excluded. As a consequence, some useful and relevant studies might have been missed, especially from non-anglophone contexts and cultures. The electronic databases PubMed, CINAHL, and PsycINFO were searched for relevant articles. These databases cover disciplines relevant to our topic such as medicine, psychology, psychiatry, nursing, behavioral sciences, and health sciences. However, there is a chance of missing relevant literature because of not including more databases, such as Scopus and Web of Science. Moreover, we restricted our study for articles published between 2010 and 2021. We wanted to conduct a comprehensive and up-to-date study as a starting point for future initiatives to offer better support to ICGs based on their current needs. Although, a few relevant studies published before 2010 may have been missed.

We also found some strengths and limitations of the literature included in this review. One strength is that most of the articles were qualitative studies, which made the data descriptive in nature. The richness of data offers an in-depth understanding of the needs of different groups of ICGs, allowing us to make a good comparison between the needs of different groups of ICGs. We also found that most of the included qualitative articles were of high quality with respect to clarity and appropriateness of study aim, qualitative methods, recruitment strategies, data collection methods, and statement of findings. However, overall, quantitative articles were a mix of moderate and weak quality. It needs to be noticed that the quality is indicated based on the information reported in the included article and not on the actual quality of the studies. A limitation is that the included studies in this systematic review are mostly conducted in western countries, except for four studies that were conducted in Asian countries, that is, Japan (Johannessen et al., 2017; Tatangelo et al., 2018), India (Amaresha et al., 2015) and Taiwan (Yang et al., 2017). Different countries represent different cultures and socio-economic statuses, which may influence the needs of these ICGs. For example, ICGs in a collectivistic society, in which the community works together and has shared goals (Darwish and Huber, 2003), may receive more support from family and friends, and therefore may report less unmet needs for support from friends and family (Pérez-Arce, 1999) as compared to ICGs in an individualistic culture, where the goals of the individuals are more oriented around the self (Darwish and Huber, 2003). However, with respect to formal support, ICGs living in higher income countries may receive more financial aid and in turn may indicate less need for financial resources as compared to lower income countries (Pérez-Arce, 1999). Although, the results of the four studies conducted in Asian countries were in line with the studies that were conducted in western origin, we have to be careful in generalizing our findings across countries.

Moreover, regardless that we explored all types of relationships in this review, we found only one article for another ICG-CR relationship, namely for parental ICGs taking care of an adult child aged 18 years or older (Minnes et al., 2010). The limited or unavailable literature for other groups of ICGs, such as, ICGs taking care of adult children or grandparents limit our findings to the groups included in this study, but highlights as well the necessity to explore the needs of other groups of caregivers in future research. In addition, there were fewer articles reporting the needs of adult child and adult sibling ICGs as compared to the spousal ICGs, and in some cases needs were expressed only in one article. For example, the need to be seen as a carer in adult sibling ICGs has been reported in only one article. Future research could focus more extensively on the needs of the adult child and adult sibling ICGs.

In our study, we included all the needs of ICGs based on their relationship with the CR except for the needs of ICGs toward an intervention or program. Although, we encountered several articles on this topic while screening the articles, therefore, for future research, it would be interesting to compare the need for interventions and programs among ICGs groups. This will contribute in enriching and supporting the literature on needs of ICGs groups. Thus, help in tailoring the interventions and programs based on the unique needs of these ICGs specifically toward an intervention.

## Conclusion and Future Implication

The synthesis of the 22 included articles resulted in seven themes of needs among spousal, adult child, and adult sibling ICGs, i.e., a need for information, support, personal time, managing personal concern such as help with managing their health, to maintain their relationship with their CR, to be seen as carer, and to be acknowledged. The three groups of ICGs represented certain common as well as unique needs. By knowing the common and unique needs of the different groups of ICGs, we can offer more targeted and personalized support to the ICGs, and design targeted interventions in the future. This might help to improve the quality of life of both the caregivers and that of their care recipient.

## Data Availability Statement

The original contributions presented in the study are included in the article/Supplementary Material, further inquiries can be directed to the corresponding author/s.

## Author Contributions

SD and GF screened the relevant article for eligibility based on the inclusion criteria. SD, AL, GF, and MH conducted the thematic analysis to synthesize the data for both qualitative and quantitative studies. SD, AL, and MH drafted the article. GF and GL commented, contributed, and edited the subsequent drafts. All authors contributed to the article and approved the submitted version.

## Conflict of Interest

The authors declare that the research was conducted in the absence of any commercial or financial relationships that could be construed as a potential conflict of interest.

## Publisher’s Note

All claims expressed in this article are solely those of the authors and do not necessarily represent those of their affiliated organizations, or those of the publisher, the editors and the reviewers. Any product that may be evaluated in this article, or claim that may be made by its manufacturer, is not guaranteed or endorsed by the publisher.
