# Supplementary material for: Exploring the Needs of Spousal, Adult Child, and Adult Sibling Informal Caregivers: A Mixed-Method Systematic Review
Source: Front Psychol. 2022 Mar 25;13:832974. doi: 10.3389/fpsyg.2022.832974 (PMC8992373; doi:10.3389/fpsyg.2022.832974)
Supplement: Supplementary file 1 [file Table_1.pdf]

Table 1. Characteristics of the included studies

Qualitative studies						
**Articles on needs of spousal ICGs**						
**Author (year), country**	**Study aim**	**Study design**	**Sample characteristics**	**Relationship of ICGs with their CR**	**Illness of CR**	**Type of needs expressed by ICGs**
**Andela, et al., (2019),**<br><br>**The Netherlands**	To elucidate the impact of the pituitary condition on the lives of partners of people with pituitary disease	Focus group study conducted with four focus groups of partners of people with pituitary disease<br><br>Data analyzed using an experiential thematic analysis	20 partners<br><br>(Mean age: 48 years)<br><br>11 women<br><br>9 men	Partner<br><br>17 married<br><br>3 living together	Pituitary disease (Cushing's disease, non-functioning adenoma, acromegaly, prolactinom)	**Unmet needs regarding care:**<br><br>- Insufficient information about the disease and its treatment<br><br>- No recognition for certain questions regarding the CR's disease, medication, and treatment<br><br>- Needed doctors to use less jargon to facilitate understanding<br><br>- Needed guidance with: (i) their own psychological issues, (ii) potential psychological symptoms of their CR, (iii) how to best support their CR<br><br>- Needed peer support

**Badr et al., (2016),**<br><br>**The USA**	To understand patients’ and spouses' **unmet needs** and relationship challenges during curative radiotherapy for head and neck cancer	Semi-structured interviews<br><br>Data analyzed using grounded theory analysis	Six spouses<br><br>(Mean age: 50.8 years)<br><br>5 women<br><br>1 man	Spouse<br><br>5 married<br><br>1unknown	Head and neck cancer (HNC)	**Unmet information needs regarding**:<br><br>- the severity of physical symptoms<br><br>- clearer timeline for CR’s recovery<br><br>**Unmet emotional and psychological needs as they:**<br><br>- felt distressed in witnessing the CR’s suffering<br><br>- faced difficulty in dealing with CR’s emotional reactions<br><br>- Cited barriers like time, competing priorities, and distance in availing support groups<br><br>**Need to improve the relationship with their CR as they:**<br><br>- had to hold back their emotions from the CR<br><br>- expressed a lack of sexual intimacy and closeness to their CR<br><br>- could not continue the social and leisure routines with their CR
**Evertsen & Wolkenstein, (2010),**	To explore the interaction of the female partner with the patient’s physicians	Focus group study conducted with two focus groups of women whose partners were	14 partners<br><br>(Mean age: 61.6 years)	Partner	Prostate cancer	**Support needs**<br><br>- Emotional support from friends, primary care physicians, urologists and

**The USA**	(primary care and urologist) and her **support needs associated with treatment of and recovery from prostate cancer**	diagnosed with prostate cancer.<br><br>Data analysis using the framework approach of familiarization, identifying thematic framework, indexing, charting, and interpretation	All women	13 married<br><br>1 unknown		support groups for themselves as well as for their CR<br><br>- Needed public figure to serve as an advocate for prostate cancer and discuss the long road after treatment<br><br>**Information needs**<br><br>Participants needed information about:<br><br>- their role as a caregiver<br><br>- how difficult and long the CR's recovery is going to be
**Habermann & Shin, (2017),**<br><br>**The USA**	To explore how couples with Parkinson's disease discuss their **needs**, concerns, and preferences **at the advanced stages of illness**	Qualitative descriptive approach using semi-structured interviews<br><br>Data analyzed thematically	14 spouses<br><br>(Mean age: 72.13 years)<br><br>7 women<br><br>7 men	Spouse<br><br>All married	Parkinson's disease (PD)	**Unmet financial needs** regarding inadequate resources and the resultant financial strain<br><br>**Respite care needs for the CR**. The needs arose from:<br><br>- not feeling that they could leave their CR due to concerns about safety<br><br>- ICGs needed a break or needed to be able to sleep

**Hupcey et al., (2011),**<br><br>**The USA**	To describe the **palliative needs** of spousal caregivers of patients followed up at specialized heart failure centers within the context of the dynamic ebb and flow of heart failure	Longitudinal study using interviews<br><br>Data analyzed using grounded theory	45 spouses<br><br>(Mean age: 60 years)<br><br>39 women<br><br>6 men	Spouses [c]	Advanced heart failure	**In times of medical instability:**<br><br>Easy understandable **information need** for-<br><br>- treatment options<br><br>- advance directives<br><br>- making decisions while the loved one is in the hospital<br><br>**In times of medical stability:**<br><br>**Information need** for-<br><br>- future planning (legal planning, advanced directives)<br><br>- new drug or treatment regimens, diet management<br><br>- making decisions about the care they were providing
**Johannessen et al., (2017),**<br><br>**Norway**	To examine the experiences and **needs for assistance** in the daily life of partners of people with young-onset frontotemporal dementia (yo-FTLD) during the	Qualitative interviews<br><br>Data analyzed by reformulated and modified method of grounded theory	16 spouses<br><br>(Mean age: 59.6 years)<br><br>9 women<br><br>7 men	Spouse<br><br>15 spouses<br><br>(9 wives<br><br>6 husbands	Young-onset frontotemporal dementia	**Assistance need through all stages of illness**<br><br>Participants needed:<br><br>- groups focusing on their needs and situations with health personnel who had competence working with yo-FTLD

	progression of the disorder			1 male cohabitant)		- daily life activities for the CR suitable to their interests, gender, and stage of life<br><br>- respite services during the evening as they needed breaks from caregiving<br><br>- day-care centers for the CR as they needed to go to work<br><br>- more information about (i) FTLD (ii) how to solve economic problems (iii) rules about services and sick leave (iv) what kinds of services were available for the CR<br><br>- health personnel to take over the responsibility for the CR and coordinate in the tasks of applications and daily services<br><br>- to get rid of the heavy administrative burden
**Le Dorze & Signori, (2010),**<br><br>**Canada**	To explore the **needs** of spouses caring for an aphasic person, and determine whether the needs were fulfilled or not. The facilitators and barriers associated with the needs	Group interviews<br><br>The data was transcribed and coded for analysis	11 spouses<br><br>(Mean age: 60.5 years)<br><br>9 women<br><br>2 men	Spouse<br><br>All married	Aphasia	**Support needs**<br><br>Participants needed:<br><br>- emotional support to deal with various consequences of aphasia<br><br>- to redefine their roles and relationship with the CR

	were also identified					- assistance in their day-to-day tasks such as cooking meals<br><br>- respite care where they wished having time for their own activities and time off from caregiving<br><br>- to improve day-to-day communication with their CR<br><br>- better interpersonal relationships with their aphasic spouse, other family members or friends<br><br>**Information needs**<br><br>Participants needed information about:<br><br>- the physical condition of their CR and aphasia itself<br><br>- public and community resources available for them<br><br>- financial resources available to them and actual financial support they may be entitled to.
**Morrisby et al., (2019),**<br><br>**Australia**	To identify **care and support needs**, as reported by people with dementia and their spousal carers living in the community in	Semi-structured interviews were conducted using an interpretive description approach followed by two focus	Interview: 10 dyadic couples<br><br>(Mean age: 74 years)[b]	Spouse [c]	Dementia	Participants needed:<br><br>- supportive and well-educated social environment<br><br>- social support at three levels: family, friends, and broader social networks

	metropolitan Western Australia	groups with spousal carers<br><br>Comparative analysis was used to develop themes regarding needs	Focus groups: 10 spouses ICGs, 3 from the interviews and 7 recruited via advocacy organisations and service providers in metropolitan Perth [a b]			- knowledge and understanding of dementia among broader social networks, such as colleagues<br><br>- access to quality services that met their needs, particularly in the moderate to late stages of dementia<br><br>- institutional support that is timely, effective and affordable<br><br>- formal community services, such as respite care, domestic assistance or financial support<br><br>- strong ongoing relationship with the service providers<br><br>- to work in partnership with their CR to continue their roles or develop new roles<br><br>- to take responsibility for looking after themselves.<br><br>**Unmet needs:**<br><br>- lack of empathy from health professionals and inconsistency in the care provided
**Wawrziczny et al., (2017),**<br><br>**France**	To explore the **needs** of spousal ICGs of persons with dementia (PWD) and then to	Semi-structured interviews	Two groups were recruited for this study:	Spouse [c]	Early and late-onset dementia	Participants needed:

	compare them based on the PWDs age at disease onset	The data was analyzed in two steps:<br><br>- the first step was qualitative to identify the needs<br><br>- the second step was quantitative to compare the needs depending on the persons with dementia's age at onset	**Group 1:**<br><br>40 spousal ICGs of early-onset of dementia (EOD) patients<br><br>(Mean age: 57.4 years)<br><br>3 women<br><br>17 men<br><br>**Group 2:**<br><br>38 spousal ICGs of late-onset of dementia (LOD) patients<br><br>(Mean age: 77.0 years)<br><br>20 women<br><br>18 men			- time to relax, release tension and take time for leisure. They wanted to have access to respite solutions<br><br>- to maintain the relationship with their CR<br><br>**Psychosocial needs**<br><br>Participants needed more visits from friends and family members, especially children<br><br>**Care-related needs**<br><br>Participants needed:<br><br>- information about the disease, treatments, and ways to react to certain behaviours of PWD<br><br>- to feel valued in their caring activities and initiatives<br><br>**Support needs**<br><br>Participants needed:<br><br>- information on existing financial material, social, or home assistance<br><br>- flexibility in the development of home help to suit the personality of the PWD
**Articles on needs of adult child ICGs**						

Author (year), country	Study aim	Study design	Sample characteristics	Relationship of ICGs with their CR	Illness of CR	Type of Needs expressed by ICGSs
**Barca et al., (2014),**<br><br>**Norway**	To explore how adult children of a parent with young-onset dementia have experienced the development of their parents' dementia and what **needs they have for assistance**	Qualitative interviews using a semi-structured guide.<br><br>A modified version of the method of "grounded theory" was applied in this study	14 adult children<br><br>(Mean age: 22.5 years)<br><br>12 women<br><br>2 men	Adult child	Young-onset dementia	**Need to be seen as a person with individual needs**<br><br>Participants needed:<br><br>- to talk about their situation and experiences with professional workers as well as with someone they can trust among their friends and family<br><br>- to make contact with the healthcare system in applying for some support for themselves<br><br>- to be followed up over time, being offered a stable contact with someone with that responsibility<br><br>- support group of their age to share their experience and identify with others<br><br>- wider audience to be informed about young onset dementia.<br><br>**Need for information**<br><br>Participants needed information about:<br><br>- the specific dementia diagnosis

						- the progression expected<br><br>- how to handle the situation for the CR, the spouse, all the family members, and themselves
**Nicholls et al., (2017),**<br><br>**The UK**	To determine whether current knowledge from cancer literature regarding young carers is generalizable to chronic conditions and, therefore, whether an existing screening tool could be adapted for this population	Face-to-face,<br><br>one-to-one interviews using interpretative phenomenological analysis	7 adult children<br><br>(Mean age: 17.9 years)<br><br>5 women<br><br>2 men	Adult child	Chronic conditions (Migraine, Arthritis, Epilepsy, diabetes, renal failure, poycyctic kidney disease, chronic fatigue syndrome)	**Need for information**<br><br>Participants needed information about:<br><br>- the condition of their CR at each stage of the condition<br><br>- the long-term implications of the CR's condition to gain personal control over the otherwise unpredictable nature of their situation, especially where there was a chance of hereditability<br><br>- educating their wider social network<br><br>**Support need**:<br><br>- they lacked family and peer support<br><br>- needed professional input like counselling services but the support was not readily available<br><br>**Need to be acknowledged as a caregiver**: Need for<br><br>acknowledgement of their role in their CR's condition by others

						**Need to be more than a carer**: Need for respite, to allow themselves to be a young adult without any unexpected responsibilities
**Articles on needs of spousal and adult child ICGs**						
**Author (year), country**	**Study aim**	**Study design**	**Sample characteristics**	**Relationship of ICGs with their CR**	**Illness of CR**	**Type of needs expressed by ICGSs**
**Figueiredo et al., (2016)**,<br><br>**Portugal**	To explore the experience of husbands and sons providing care to a family member with moderate-to severe chronic obstructive pulmonary disease (COPD)	A cross-sectional qualitative study using in-depth, semi-structured individual interviews	7 husbands<br><br>(Mean age: 70.9 years)<br><br>5 sons<br><br>(Mean age: 43.4 years)<br><br>All men	Spouse (husband) and adult child (son)	Chronic obstructive pulmonary disease (COPD)	**Husbands**<br><br>Participants needed:<br><br>- to learn practical skills to manage COPD and adequate treatment<br><br>- to improve their ability to care. Specifically, the need to respond appropriately to the worsening of symptoms<br><br>**Sons:**<br><br>Participants needed:<br><br>- more information to (i) become aware of the disease severity, (ii) deal

						with the care receiver's breathlessness, and (iii) cope better with the disease<br><br>- self-confidence in providing care as they felt insecure about providing personal care
**Tatangelo et al., (2018),**<br><br>**Australia**	To examine the **health needs** of partner and offspring caregivers of older people with dementia, including the barriers they experience in meeting their needs	Semi-structured interviews<br><br>Transcribed and analyzed using thematic analysis	12 partners<br><br>(Mean age: 65.7 years)<br><br>7 women<br><br>4 men;<br><br>12 adult children<br><br>(Mean age: 51.1 years)<br><br>10 women<br><br>2 men	Partner [c] and adult child	Dementia	**Common needs:**<br><br>- Unmet health needs across several areas including exercise, social relationships, mental health and having time for themselves<br><br>**Different needs:**<br><br>- Partner caregivers demonstrated unmet emotional support needs, as they no longer had emotional support from their partner with dementia. They had an unmet need for time away from the caregiving role<br><br>- Offspring caregivers' unmet health needs were focused on their unmet mental health needs and feeling socially isolated
**Articles on needs of sibling ICGs**						

**Arnold et al., (2012),**<br><br>**The USA**	To investigate the support needs of siblings of people with a developmental disability so they can be addressed by parents, professionals, and policymakers	The Supporting Siblings Survey which included the open-ended questions capturing the descriptive information about the concerns and the support needs of siblings	139 adult siblings<br><br>(Mean age: 37 years)<br><br>120 women<br><br>10 men	Adult sibling	Developmental disability	**Need to be included in supports and services**<br><br>Participants needed:<br><br>- group support with other siblings to enable open conversations about their situation. They felt neglected and desperately wanted to be included.<br><br>- more adult sibling groups as people tend to forget about siblings specially when they are adult<br><br>**Need to be a part of services and support available for the parents**<br>They wanted to have their voice heard and be treated as people with a valuable role and perspective<br><br>**Need for information and education**<br><br>Participants needed:<br><br>- education and training opportunities such as conferences, workshops, and seminars<br><br>- siblings and parents to go through the future family plans together<br><br>- programs that explain how to get services and what services are provided for the siblings

						- general public to be educated about the people with disability<br><br>**Need regarding the formal disability service system**<br><br>Participants needed:<br><br>- great improvement in the system to better support their entire family as well as siblings.<br><br>- funding sources and financial support<br><br>- Respite services were to give families ‘‘a break’’ and have time apart from the person with a disability to do other tasks and rest
**Amaresha et al., (2015),**<br><br>**India**	To explore the needs of the siblings of persons with schizophrenia with regard to the care taking of patients suffering from schizophrenia attending a tertiary care mental health hospital in Southern India	Qualitative explorative study used semi-structured face-to-face interviews for data collection and adopted a general inductive approach for data analysis	15 siblings<br><br>(Mean age: 33 years)<br><br>4 women<br><br>11 men	Adult sibling	Schizophrenia	**Need for psychosocial**<br><br>Participants needed:<br><br>- help of mental health professionals in managing the behavior of the affected sibling at home.<br><br>- help in managing side effects of medications, and help the affected sibling cope up while they experience hallucinations and other problematic behaviors<br><br>**Need for follow-up services**<br><br>Participants needed:

						- brief sessions during the follow-ups of their affected siblings.<br><br>- telephonic consultations in case of emergency and for any other clarifications<br><br>**Need for information**<br><br>-Needed information on causes, prognosis, treatment related information, side effects etc.<br><br>**Personal needs**<br><br>Participants needed help in:<br><br>- maintaining healthy communication with the ill siblings.<br><br>- managing day-to-day stressors.<br><br>- addressing their concerns related to fear of heredity<br><br>**Miscellaneous needs**<br><br>Participants needed:<br><br>- help in accessing welfare benefits such as free medications and bus pass.<br><br>- more attention from professionals to address patients’ needs and home visits

**Davys et al., (2016),**<br><br>**The UK**	To explore the perceptions of siblings of adults who have an intellectual disability regarding future wishes and family expectation of future support	Face-to-face semi-structured interviews<br><br>An approach aligned to Interpretative<br><br>Phenomenological Analysis (IPA) was used to analyze the data	15 siblings [a]<br><br>12 women<br><br>3 men	Adult sibling	Intellectual disability	Participants needed:<br><br>- detailed advice and information<br><br>- emotional support for mental health issues<br><br>- help with practical solutions<br><br>- to be seen as having a separate identity<br><br>- to look after their own health, well-being and avoid self-blame
**Grant et al., (2021),**<br><br>**The USA**	To identify the perceptions and **support needs** of siblings, who often have lifelong relationships and assume important roles for their brothers and sisters with mucopolysaccharidoses (MPS)	A survey collecting both quantitative and qualitative information through a series of 17 Likert statements and five open-ended question respectively. The questions regarding needs were asked only through two open ended questions	97 siblings [a]<br><br>83 women<br><br>14 men	Adult sibling	Mucopolysaccharidoses (MPS)	**Need for support**<br><br>Participants needed:<br><br>- parents and professionals to acknowledge and support them<br><br>- to speak and connect with other siblings who have brothers or sisters with MPS<br><br>- support from both in-person (e.g. workshops) and online (e.g. forums) groups would be helpful<br><br>- opportunities to be included in medical conversations and care plans for their CR

						- doctors to provide information about MPS to them
**Yang et al., (2017),**<br><br>**China**	To understand siblings' experiences as the primary caregiver of patients with schizophrenia in Taiwan	Qualitative descriptive approach using semi-structured in-depth interviews<br><br>Qualitative content analysis was used to analyze the data	10 siblings<br><br>(Mean age: 44.9 years)<br><br>7 women<br><br>3 men	Adult sibling	Schizophrenia	**Need for support**<br><br>Needed support from multiple sources, such as their family of origin, their nuclear family, and the mental health-care system, especially given the challenges and difficulties of taking on caregiving responsibility for a person with schizophrenia
**Quantitative studies**						
**Articles on needs of spousal ICGs**						
**Author (year),**<br><br>**country**	**Aim of the study**	**Study design**	**Sample characteristics**	**Relationship of ICGs with their CR**	**Illness of CR**	**Type of needs expressed by ICGs**
**Kobayakawa et al., (2016),**<br><br>**Japan**	To understand the **need for psychological support from mental health specialists** among bereaved family members who care	A multicenter cross-sectional survey developed by the authors based on literature review, interviews, and focus groups with stakeholders	360 spousal ICGs[a,b]	Spouses [c]	Cancer	Needed psychological support from mental health specialists

	for patients with cancer					
**Turner et al., (2013),**<br><br>**The UK**	To study the health status, levels of anxiety and depression, **unmet supportive care needs** and positive outcomes in the partners/family members of breast, prostate and colorectal cancer survivors 5–16 years post diagnosis	Cross-sectional study using a self-administered Cancer Survivors' Partners Unmet Needs (CaSPUN) questionnaire	212 partners and spouses<br><br>(Mean age: 65.5 years) [b]	Partner<br><br>109 husbands<br><br>96 wives 7 partners	Breast, prostate and colorectal cancer	**Nine most common unmet needs:**<br><br>**Participants needed**<br><br>- more accessible hospital parking<br><br>- help to manage their concerns about the cancer coming back<br><br>- to know that their partner's doctors talk to each other to<br><br>co-ordinate care<br><br>- help getting life and/or travel insurance<br><br>- to feel like they are managing their as well as their partner's health together with the medical team<br><br>- help to find out about financial support and/or government benefits to which they are entitled<br><br>- an ongoing single contact to whom they can go to find out about services whenever needed<br><br>- local health care services that are available when they or their partner needs them

						- for any complaints regarding their or their partner's care to be properly addressed
**Articles on needs of adult child ICGs**						
**Author (year), country**	**Study aim**	**Study design**	**Sample characteristics**	**Relationship of ICGs with their CR**	**Illness of CR**	**Type of Needs expressed by ICGSs**
**Veil et al., (2013),**<br><br>**Australia**	To explore the expectations, **needs** and concerns of adult children about their elderly parents	Cross-sectional survey study having open ended questions<br><br>The data was analyzed thematically	68 adult children [a] [b]	Adult child	Frailty	Needed better information. This included information about their parent's entitlements, access to services and support, and knowing where to go for support
**Articles on needs of spousal and adult child ICGs**						
**Author (year), country**	**Study aim**	**Study design**	**Sample characteristics**	**Relationship of ICGs with their CR**	**Illness of CR**	**Type of Needs expressed by ICGSs**

**Peeters et al., (2010),**<br><br>**The Netherlands**	To understand the **needs** of informal caregivers **for additional professional support**, while also discussing the professional support they already receive	Survey study using a questionnaire developed by the Netherlands Institute for Health Care Services Research (NIVEL)	490 spouses [a b] and 372 adult children [a b]	Spouse [c] and adult child (sons/ daughters, and sons/daughters in law)	Dementia	**Common needs:**<br><br>Needed professional support, but there were significant differences between the spousal and adult child ICGs with respect to the type of professional support:<br><br>**Different needs:**<br><br>- spousal ICGs more often needed emotional support<br><br>- adult child ICGs more often needed information and coordination of dementia care
