# Supplementary material for: Exploring the Needs of Spousal, Adult Child, and Adult Sibling Informal Caregivers: A Mixed-Method Systematic Review
Source: Front Psychol. 2022 Mar 25;13:832974. doi: 10.3389/fpsyg.2022.832974 (PMC8992373; doi:10.3389/fpsyg.2022.832974)
Supplement: Supplementary file 2 [file Table_2.pdf]

Appendix A

***PRESS Guideline* — Search Submission & Peer Review Assessment**

SEARCH SUBMISSION: THIS SECTION TO BE FILLED IN BY THE SEARCHER

Searcher: Srishti Dang	Email: s.dang@umcg.nl	
Date submitted: 19-11-2019	Date requested: 27.11.2019	*[Maximum = 5 working days]*

Systematic Review Title:

Identifying the needs of informal caregivers based on their relationship with the care recipient – A mixed method systematic review.

Revised:

Exploring the needs of spousal, adult child and adult sibling informal caregivers: A mixed-method systematic review

This search strategy is …

Yes	My PRIMARY (core) database strategy — First time submitting a strategy for search question and database
	My PRIMARY (core) strategy — Follow-up review NOT the first time submitting a strategy for search question and database. If this is a response to peer review, itemize the changes made to the review suggestions
	SECONDARY search strategy— First time submitting a strategy for search question and database
	SECONDARY search strategy — NOT the first time submitting a strategy for search question and database. If this is a response to peer review, itemize the changes made to the review suggestions

Database

(i.e., MEDLINE,CINAHL…):

MEDLINE, PsycINFO, CINAHL *[mandatory]*

Interface

(i.e., Ovid, EBSCO…):

MEDLINE with PubMed, PsycINFO and CINAHL with EBSCO *[mandatory]*

Research Question

(Describe the purpose of the search) *[mandatory]*

Research question: What are the needs of informal caregivers based on their relationship with the care recipient (e.g., caregivers taking care of a spouse, parent, child or sibling)?

Purpose of the search: Through this study, we will outline the needs of different groups of informal caregivers based on the relationship they have with the care recipient.

PICO Format

(Outline the PICOs for your question — i.e., Patient, Intervention, Comparison, Outcome, and Study Design — as applicable)

**P**	Informal Caregivers: people who take care of a family member in case of any mental or physical illness, or disability.
**I**	N/A
**C**	Comparison of the needs of different informal caregivers taking care of an adult family member (e.g., informal caregivers taking care of a spouse, adult child, parent or adult sibling).
**O**	The needs of different informal caregivers.
**S**	Qualitative, quantitative and mixed method studies.

Inclusion Criteria

(List criteria such as age groups, study designs, etc., to be included) *[optional*

Studies with -

- the needs/unmet needs of different informal caregivers, e.g. - e.g., informal caregivers taking care of a spouse, adult child, parent or an adult sibling.
- qualitative, quantitative or mixed-method study design.
- Articles from the year 2019-2010
- publication in English

Exclusion Criteria

(List criteria such as study designs, date limits, etc., to be excluded) *[optional]*

- Studies in which the data on the needs of different informal caregivers groups could not be analyzed separately for different groups of caregivers.
- Grey literature i.e., conference abstracts, presentations, proceedings; regulatory data; unpublished trial data; government publications; reports (such as white papers, working papers, internal documentation).

Was a search filter applied? NO

Yes No

If YES, which one(s) (e.g., Cochrane RCT filter, PubMed Clinical Queries filter)? Provide the source if this is a published filter. *[mandatory if YES to previous question — textbox]*

Other notes or comments you feel would be useful for the peer reviewer? ***[optional]***

The search string was generated and different database were searched with the help of the librarian of the Health Psychology department of the UMCG, Truus van Ittersum.

Please copy and paste your search strategy here, exactly as run, including the number of hits per line. ***[mandatory]***

Pubmed:

Line 1 (Informal caregiving population):

("Caregivers"[Mesh] OR caregiv*[tiab] OR (("Family"[Mesh] OR family[tiab] OR spous*[tiab] OR parent*[tiab] OR husband*[tiab] OR wife[tiab] OR wives[tiab] OR partner*[tiab] OR adult child*[tiab]) AND caring[tiab]))

AND

Line 2 (needs)

("Health Services Needs and Demand"[Mesh] OR "Needs Assessment"[Mesh] OR needs[tiab])

AND

Line 3 (family relations):

("Spouses"[Mesh] OR "Siblings"[Mesh] OR wife[tiab] OR wives[tiab] OR husband*[tiab] OR marital[tiab] OR spous*[tiab] OR Partner*[tiab] OR Couple*[tiab] OR sibling*[tiab] OR adult child*[tiab] OR brother*[tiab] OR sister*[tiab] OR daughter*[tiab] OR son[tiab] OR sons[tiab] OR granddaughter*[tiab] OR grandson*[tiab] OR grandchild*[tiab])

Combi:

(((("Caregivers"[Mesh] OR caregiv*[tiab] OR (("Family"[Mesh] OR family[tiab] OR spous*[tiab] OR parent*[tiab] OR husband*[tiab] OR wife[tiab] OR wives[tiab] OR partner*[tiab] OR Couple*[tiab] OR adult child*[tiab]) AND caring[tiab])))) AND (("Health Services Needs and Demand"[Mesh] OR "Needs Assessment"[Mesh] OR needs[tiab]))) AND (("Spouses"[Mesh] OR "Siblings"[Mesh] OR wife[tiab] OR wives[tiab] OR husband*[tiab] OR marital[tiab] OR spous*[tiab] OR sibling*[tiab] OR adult child*[tiab] OR brother*[tiab] OR sister*[tiab] OR daughter*[tiab] OR son[tiab] OR sons[tiab] OR granddaughter*[tiab] OR grandson*[tiab] OR grandchild*[tiab]))

PsycINFO

Line 1 (Informal caregiving population):

(DE "Caregivers" OR (TI caregiv* OR AB caregiv* OR KW caregiv*)) OR (((DE "Family" OR DE "Biological Family" OR DE "Dual Careers" OR DE "Dysfunctional Family" OR DE "Extended Family" OR DE "Family Background" OR DE "Family History" OR DE "Family Members" OR DE "Family Relations" OR DE "Family Resemblance" OR DE "Family Structure" OR DE "Family Work Relationship" OR DE "Family of Origin" OR DE "Interethnic Family" OR DE "Interracial Family" OR DE "Military Families" OR DE "Nepotism" OR DE "Nuclear Family" OR DE "Schizophrenogenic Family" OR DE "Stepfamily") OR TI (family OR spous* OR parent* OR husband* OR wife OR wives OR partner* OR Couple* OR adult child*) OR AB (family OR spous* OR parent* OR husband* OR wife OR wives OR partner* OR adult child*) OR KW (family OR spous* OR parent* OR husband* OR wife OR wives OR partner* OR adult child*)) AND (TI caring OR AB caring OR KW caring))

Line 2 (needs)

(DE "Needs" OR DE "Health Service Needs" OR DE "Psychological Needs" OR DE "Special Needs" OR DE "Needs Assessment") OR (TI needs OR AB needs OR KW needs)

Line 3 (family relations):

(DE "Couples" OR DE "Same Sex Couples" OR DE "Spouses" OR DE "Husbands" OR DE "Wives" OR DE "Siblings" OR DE "Brothers" OR DE "Multiple Births" OR DE "Sisters") OR (TI (wife OR wives OR husband* OR marital OR spous* OR sibling* OR adult child* OR brother* OR sister* OR daughter* OR son OR sons OR granddaughter* OR grandson* OR grandchild*) OR AB (wife OR wives OR husband* OR marital OR spous* OR sibling* OR adult child* OR brother* OR sister* OR daughter* OR son OR sons OR granddaughter* OR grandson* OR grandchild*) OR KW (wife OR wives OR husband* OR marital OR spous* OR sibling* OR adult child* OR brother* OR sister* OR daughter* OR son OR sons OR granddaughter* OR grandson* OR grandchild*))

Combi:

((DE "Caregivers" OR (TI caregiv* OR AB caregiv* OR KW caregiv*)) OR (((DE "Family" OR DE "Biological Family" OR DE "Dual Careers" OR DE "Dysfunctional Family" OR DE "Extended Family" OR DE "Family Background" OR DE "Family History" OR DE "Family Members" OR DE "Family Relations" OR DE "Family Resemblance" OR DE "Family Structure" OR DE "Family Work Relationship" OR DE "Family of Origin" OR DE "Interethnic Family" OR DE "Interracial Family" OR DE "Military Families" OR DE "Nepotism" OR DE "Nuclear Family" OR DE "Schizophrenogenic Family" OR DE "Stepfamily") OR TI (family OR spous* OR parent* OR husband* OR wife OR wives OR partner* OR Couple* OR adult child*) OR AB (family OR spous* OR parent* OR husband* OR wife OR wives OR partner* OR adult child*) OR KW (family OR spous* OR parent* OR husband* OR wife OR wives OR partner* OR adult child*)) AND (TI caring OR AB caring OR KW caring))) AND ((DE "Needs" OR DE "Health Service Needs" OR DE "Psychological Needs" OR DE "Special Needs" OR DE "Needs Assessment") OR (TI needs OR AB needs

OR KW needs)) AND ((DE "Couples" OR DE "Same Sex Couples" OR DE "Spouses" OR DE "Husbands" OR DE "Wives" OR DE "Siblings" OR DE "Brothers" OR DE "Multiple Births" OR DE "Sisters") OR (TI (wife OR wives OR husband* OR marital OR spous* OR sibling* OR adult child* OR brother* OR sister* OR daughter* OR son OR sons OR granddaughter* OR grandson* OR grandchild*) OR AB (wife OR wives OR husband* OR marital OR spous* OR sibling* OR adult child* OR brother* OR sister* OR daughter* OR son OR sons OR granddaughter* OR grandson* OR grandchild*) OR KW (wife OR wives OR husband* OR marital OR spous* OR sibling* OR adult child* OR brother* OR sister* OR daughter* OR son OR sons OR granddaughter* OR grandson* OR grandchild*)))

CINAHL:

Line 1 (Informal caregiving population):

((MH "Caregivers") OR (TI caregiv* OR AB caregiv*)) OR (((MH "Family+") OR (TI (family OR family OR spous* OR parent* OR husband* OR wife OR wives OR partner* OR adult child*) OR AB (family OR family OR spous* Partner* OR Couple* OR parent* OR husband* OR wife OR wives OR adult child*))) AND (TI caring OR AB caring))

Line 2 (needs)

(MH "Health Services Needs and Demand+" OR MH "Needs Assessment" OR TI needs OR AB needs)

Line 3 (family relations):

(MH "Spouses") OR (MH "Siblings") OR TI (wife OR wives OR husband* OR marital OR spous* OR sibling* OR adult child* OR brother* OR sister* OR daughter* OR son OR sons OR granddaughter* OR grandson* OR grandchild*) OR AB (wife OR wives OR husband* OR marital OR spous* OR sibling* OR adult child* OR brother* OR sister* OR daughter* OR son OR sons OR granddaughter* OR grandson* OR grandchild*)

Combi:

(((MH "Caregivers") OR (TI caregiv* OR AB caregiv*)) OR (((MH "Family+") OR (TI (family OR family OR spous* OR parent* OR husband* OR wife OR wives OR partner* OR adult child*) OR AB (family OR family OR spous* OR Partner* OR Couple* OR parent* OR husband* OR wife OR wives OR adult child*))) AND (TI caring OR AB caring))) AND ((MH "Health Services Needs and Demand+" OR MH "Needs Assessment" OR TI needs OR AB needs)) AND ((MH "Spouses") OR (MH "Siblings") OR TI (wife OR wives OR husband* OR marital OR spous* OR sibling* OR adult child* OR brother* OR sister* OR daughter* OR son OR sons OR granddaughter* OR grandson* OR grandchild*) OR AB (wife OR wives OR husband* OR marital OR spous* OR sibling* OR adult child* OR brother* OR sister* OR daughter* OR son OR sons OR granddaughter* OR grandson* OR grandchild*))
